# Supplementary material for: Dynalign II: common secondary structure prediction for RNA homologs with domain insertions
Source: Nucleic Acids Res. 2014 Nov 21;42(22):13939–48. doi: 10.1093/nar/gku1172 (PMC4267632; doi:10.1093/nar/gku1172)
Supplement: SUPPLEMENTARY DATA [file supp_gku1172_nar-02021-z-2014-File012.zip › manual/Text/ProbKnot.html]

RNAstructure Command Line Help: ProbKnot


|  |  |  |
| --- | --- | --- |
|  | RNAstructure Command Line Help ProbKnot | - Contents - Index |
| ProbKnot is used to predict a maximum expected accuracy structure that can contain pseudoknots. USAGE: ProbKnot <input file> <ct file> [options] Required parameters: <input file> \| The name of a file containing input data. This input data can be in one of two formats: 1. Partition function save file (holds base pairing probability data for all pairs and can be generated using the partition interface). 2. Sequence file (holds raw sequence: .seq or .fasta). Note that lowercase nucleotides are forced single-stranded in structure prediction. Note that in order to use a squence file, the "sequence" flag must be specified (see "--sequence" below).; <ct file> \| The name of a CT file to which output will be written. Options which don't require added values: -d, -D, --DNA \| This flag only matters if the input file is a squence file and has been identified as such (see "--sequence" below). Specify that the sequence is DNA, and DNA parameters are to be used. Default is to use RNA parameters.; -h, -H, --help \| Display the usage details message.; --sequence \| Identify the input file format as a sequence file. Options which require added values: -i, -I, --iterations \| Specify the number of iterations the calculation will undergo. More iterations can result in more predicted pairs, although a single iteration is generally sufficient. Default is 1 iteration.; -m, -M, --minimum \| Specify the minimum length accepted for a helix. A post-processing filter removes short helices. Default is 3 base pairs. References: 1. Reuter, J.S. and Mathews, D.H. "RNAstructure: software for RNA secondary structure prediction and analysis." *BMC Bioinformatics*, 11:129. (2010). 2. Bellaousov, S., and Mathews, D.H. "ProbKnot: fast prediction of RNA secondary structure including pseudoknots." *RNA*, 16:1870-1880. (2010). | | |
| Visit The Mathews Lab RNAstructure Page for updates and latest information. | | |
